# Clinical management of intrahepatic cholangiocarcinoma: surgical approaches and systemic therapies

**DOI:** 10.3389/fonc.2024.1321683

**Published:** 2024-01-24

**Authors:** Samantha M. Ruff, Timothy M. Pawlik

**Affiliations:** Department of Surgery, The Ohio State University Wexner Medical Center and James Comprehensive Cancer Center, Columbus, OH, United States

**Keywords:** intrahepatic cholangiocarcinoma, surgery, FGFR2, IDH, targeted therapy

## Abstract

Intrahepatic cholangiocarcinoma (ICCA) is a rare and aggressive malignant tumor that arises from the biliary tracts in the liver. Upfront surgery with adjuvant capecitabine in patients with resectable disease is often the standard treatment. Unfortunately, only 20% of patients present with resectable disease and many individuals will develop recurrence or metastatic disease after curative-intent resection. Patients with advanced or metastatic ICCA often require multidisciplinary care with a combination of cytotoxic chemotherapy, targeted therapy, and/or locoregional therapies. Gemcitabine plus cisplatin is currently first line therapy for advanced or metastatic ICCA. In recent years, efforts have been focused to develop more effective targeted therapy, most commonly with FGFR and IDH inhibitors for ICCA. Despite these efforts, ICCA still carries a poor prognosis. We herein review the current clinical management of ICCA focusing on surgical technique and systemic therapies.

## Introduction

**Figure 1:**
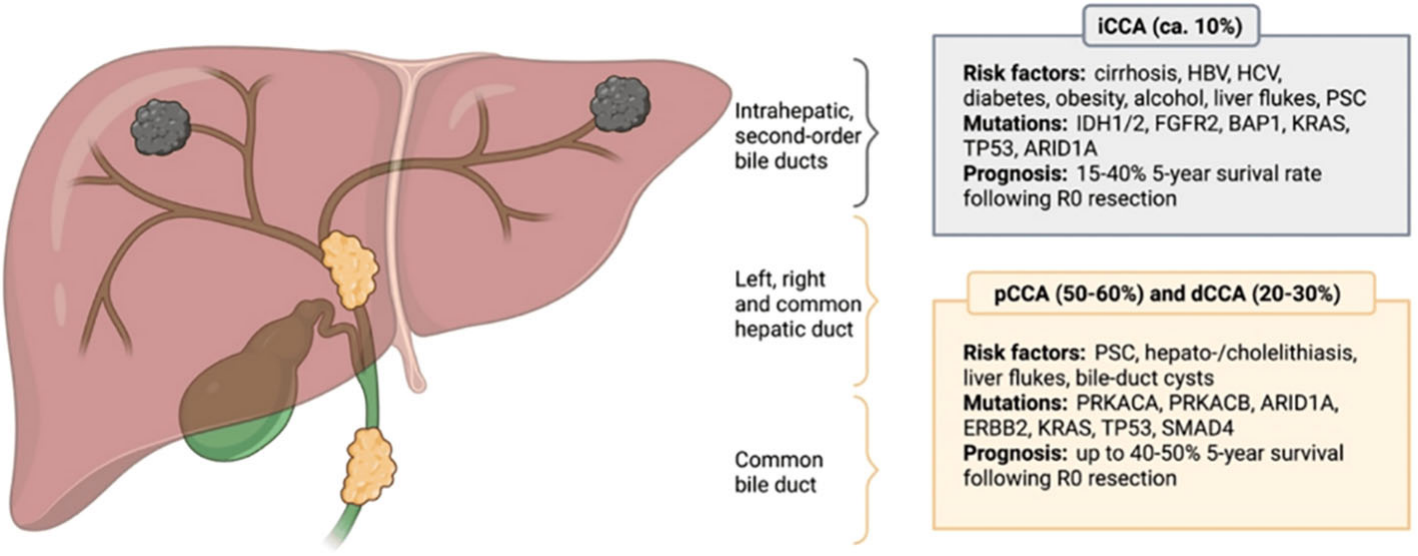
Anatomical classification of cholangiocarcinoma. CCA is anatomically divided into intrahepatic (iCCA), perihillar (pCCA) and distal (dCCA) cholangiocarcinoma, with pCCA and dCCA being summarized as extrahepatic cholangiocarcinoma (eCCA). Different CCA subtypes possess distinct molecular aberrations and differ in terms of their etiology, while certain risk factors and genetic mutations are not subtype-specific. The most common risk factors and prevailing genetic alterations are presented. *HBV,* Hepatitis B virus; *HCV,* Hepatitis C virus; *PSC,* Primary sclerosing cholangitis; *IDH1/2,* Isocitrate dehydrogenase 1/2; *FGFR2,* Fibroblast growth factor receptor 2; *BAP1,* BRCA1 associated protein 1; *KRAS,* Kirsten rat sarcoma virus; *TP53,* Tumor suppressor protein 53; *ARID1A,* AT-rich interactive domain-containing protein 1A; *PRKACA,* Protein kinase cAMP-activated catalytic subunit alpha; *PRKACB,* Protein kinase cAMP-activated catalytic subunit beta; *ERBB2,* Erb-B2 receptor tyrosine kinase 2; *SMAD4,* Mothers against decapentaplegic homolog 4. Reprinted with permission from reference (2).

Cholangiocarcinoma (CCA) is a rare and aggressive malignant tumor that arises from the biliary tracts. It is defined by its anatomic location: intrahepatic CCA (ICCA) arises from the biliary ducts in the liver and extrahepatic CCA (ECCA) arises from either the common hepatic duct (also known as a hilar or Klatskin tumor) or the distal common bile duct (1). Given the differences in incidence, risk factors, treatment response, and prognosis between ICCA and ECCA, these tumors should be considered biologically different cancers (**Figure 1**) (3).

For ICCA, upfront resection with adjuvant capecitabine in patients with resectable disease is generally the standard treatment. Optimal surgical technique is crucial to oncologic success. Unfortunately, only 20% of patients present with resectable disease and many individuals will develop recurrence or metastatic disease after curative-intent resection (4–6). Patients with advanced, recurrent, or metastatic ICCA often require multidisciplinary care with a combination of cytotoxic chemotherapy, targeted therapy, and/or locoregional therapies. Gemcitabine plus cisplatin is currently the first line therapy for advanced or metastatic ICCA. In recent years, efforts have been focused on developing effective targeted therapy, most commonly for FGFR and IDH inhibitors for ICCA (7, 8). Despite these efforts, ICCA still carries a poor prognosis. We herein review the current clinical management of ICCA focusing on surgical techniques and systemic therapies.

## Surgery for ICCA

Surgical technique, patient selection, consideration of anatomic versus non-anatomic resection, margin status, and adequate lymphadenectomy are critical elements to curative-intent resection. The imaging modalities of choice to determine resectablitity for intrahepatic cholangiocarcinoma is generally either contrast enhanced computed tomography (CT) or magnetic resonance imaging (MRI). With MRI, a magnetic resonance cholangiopancreatography (MRCP) can also be performed to better delineate the bile duct anatomy. For many patients, a successful surgery is the major contributing factor to their overall survival.

### Guideline criteria for upfront resection

According to National Comprehensive Cancer Network (NCCN) guidelines and European Association of the Study of Liver (EASL) guidelines, patients with a solitary lesion, anatomically resectable cancer, no severe comorbidities, and adequate future liver remnant (FLR) should be considered candidates for surgery (9, 10). The Association for the Study of Liver Disease (AASLD) recommends a FLR >30% in the absence of cirrhosis and >40% in patients with cirrhosis to prevent postoperative hepatic insufficiency (11–14). If there is concern for a suboptimal FLR, patients can undergo portal vein embolization to accelerate hypertrophy of the FLR (11, 15). EASL does not recommend upfront resection for patients with centrally located tumors that involve the bilateral second order bile ducts, have unilateral liver atrophy with contralateral biliary or vascular involvement, or bile duct infiltration with contralateral vascular involvement (16). Additionally, vascular resection can be considered if an R0 resection margin is achieved as per EASL guidelines (16). Advanced cirrhosis or extrahepatic disease are absolute contraindications to resection (17, 18).

### Anatomic versus non-anatomic resection

For hepatocellular carcinoma an anatomic resection that removes the tumor-bearing portal branches is preferred and may provide a survival benefit (19). However, this surgical approach has not been associated with improved outcomes among patients with ICCA. EASL guidelines recommend that non-anatomic resection be reserved for patients with small, peripheral lesions in a single segment. If more than one liver segment is involved, the guidelines recommend an anatomic resection (16, 20). Zhang et al. compared short and long-term outcomes in 1,023 patients with ICCA who underwent major or minor hepatectomy (19). Patients who underwent a major hepatectomy had a higher risk of postoperative complications, but in the propensity-matched analysis there was no difference in overall survival or recurrence free survival. In contrast, Si et al. reported different results comparing patients with ICCA who underwent anatomic versus non-anatomic resection (21). The two cohorts had a similar risk of post-operative complications, but patients who underwent an anatomic resection had better disease free and overall survival. The survival benefit was primarily seen in patients with stage IB or stage II (without microvascular invasion) disease. These data suggested that there may be a subset of patients who benefit from an anatomic resection. For now, it is acceptable to do a non-anatomic resection for patients with ICCA and the decision should be based on the tumor’s clinicopathologic factors, anatomic considerations, and future liver remnant.

### Margin status

Achieving an R0 margin should be the goal of any curative-intent resection. Achieving an R0 margin can be challenging for ICCA due to the size and location of many tumors. A study of 583 patients with ICCA treated at major hepatopancreatobiliary centers noted that 16% of patients had an R1 resection (22). These patients had a higher risk of recurrence and shorter overall survival. Furthermore, the study evaluated whether the R0 margin width contributed to long term outcomes. The authors reported that a wider R0 margin width (5-9 mm compared to 1-4 mm) was associated with better recurrence free and overall survival. However, the impact of R0 margin status width on survival reported in this study may just represent how tumor biology contributes to long term outcomes. Patients with more aggressive tumors (e.g., larger, bilateral, perineural invasion) are more likely to have an R1 margin or closer R0 margin. The International ICCA Study Group performed a retrospective study of 1,105 patients with ICCA. This group evaluated the significance of overall tumor burden and its association with margin status (23). Patients with low or medium tumor burden had improved survival as margin width increased. However, surgical margin status did not confer the same survival advantage in patients with high tumor burden. These data suggested that an R0 margin cannot overcome aggressive tumor biology.

### Lymphadenectomy

Like other cancers, lymphadenectomy provides prognostic information for patients and guides treatment decisions. As such, an assessment of the nodal basin should be done during ICCA resection. Based on the National Comprehensive Cancer Network guidelines and American Joint Committee on Cancer Staging Manual, a lymphadenectomy with at least six nodes should be performed to stage ICCA adequately (17, 24). Traditionally, a lymphadenectomy should be performed of the peri-portal lymph nodes (station 12) in addition to other nodal basins based on anatomic location of the tumor (25). Left sided ICCA should have sampling from the hepatoduodenal ligament, inferior phrenic, and gastrohepatic nodal basins, while right sided ICCA should have sampling from the hepatoduodenal ligament, peri-duodenal, and peri-pancreatic basins (10). Patients with lymph node metastases outside of station 12 tend to have worse overall survival (10). Despite these recommendations, multiple studies have demonstrated that only about half of patients who undergo ICCA resection have examination of at least one lymph node and that only about 15% of patients have the recommended six nodes identified on pathology (26, 27).

### Multifocal disease

The treatment of multifocal ICCA remains controversial. There is likely a subset of patients with multifocal disease who will benefit from resection, but it remains unclear which patients gain the most oncologic benefit from hepatectomy in this clinical setting. Patients with multifocal disease and no lymph node involvement are considered to potential benefit, even though these individuals have a worse prognosis than patients with solitary tumors. At the same time, patients with multifocal disease have a better prognosis than patients with extrahepatic metastases (17). As such, the European Network for the Study for Cholangiocarcinoma have proposed adding a new M1a stage to include patients with multifocal disease (28). Similar to existing guidelines, patients with multifocal ICCA at a multidisciplinary tumor board for consideration of upfront surgery versus neoadjuvant chemotherapy (16). Response or progression on neoadjuvant chemotherapy can provide insight into the tumor biology and inform decisions on whether patients should proceed to surgery.

### Hepatic artery infusion pump

The HAIP is a locoregional treatment that delivers chemotherapy directly to the liver through the hepatic artery. This approach allows for preferential delivery to the cancer cells prior to entering systemic circulation, which decreases the toxic side effects (29). The HAIP has primarily been employed in the treatment of metastatic colorectal cancer, but has recently demonstrated efficacy in ICCA. Floxuridine is the traditional chemotherapy used in the HAIP. Franssen et al. reported no difference in overall or progression free survival between patients with multifocal ICCA who underwent resection versus HAIP (29). These authors did note that 30 day postoperative mortality was higher in the resection cohort. In a separate retrospective study of patients with multifocal ICCA, intra-arterial therapy (transarterial chemoembolization (TACE), transarterial embolization (TAE), or HAIP) was compared with resection (30). When patients who underwent HAIP were compared directly to resection, there was improved overall survival (39 months versus 20 months, respectively). A separate phase II trial treated 38 patients with unresectable ICCA with HAIP and systemic gemcitabine and oxaliplatin (31). The ORR was 58% and DCR was 84%. Four of patients were able to be downstaged and undergo resection. HAIP will require further studies to elucidate its potential as a locoreginal therapy for ICCA but may be appropriate to control disease growth and downstage selected patients for resection.

## Systemic therapy

### Cytotoxic chemotherapy in advanced or metastatic ICCA

For patients with advanced or metastatic ICCA, gemcitabine plus cisplatin is the first line therapy. The ABC-02 phase III clinical trial compared the use of gemcitabine plus cisplatin to gemcitabine alone in 410 patients with advanced or metastatic CCA, gallbladder cancer, or ampullary cancer (32). The gemcitabine plus cisplatin cohort had improved overall survival versus gemcitabine alone cohort (median overall survival 11.7 months versus 8.1 months, p<0.001, respectively). Progression free survival in the gemcitabine plus cisplatin and gemcitabine alone cohorts was 8 and 5 months, respectively. While this trial was completed over a decade ago, gemcitabine plus cisplatin remains first line therapy for advanced ICCA.

Recent data have suggested that the addition of abraxane (also known as nab-paclitaxel) may deplete surrounding stromal tissue and improve the delivery of gemcitabine (33, 34). Pre-clinical studies in pancreatic cancer have demonstrated that nab-paclitaxel results in the reduction of α-smooth muscle actin and collagen 1 expression in murine models and a depletion of desmoplastic stroma in patient derived xenograft models. These findings were accompanied by increased vascularization and subsequent delivery of gemcitabine (34, 35). One pre-clinical study demonstrated that nab-paclitaxel altered stromal fibrosis of human peritoneal mesothelial cells through suppression of TGF-β/SMAD signaling pathway. While this mechanism is still poorly understood, it is hypothesized that nab-paclitaxel is bound to albumin and therefore has enhanced drug delivery to the tumor (36). In examining resected pancreatic adenocarcinoma specimen after neoadjuvant nab-paclitaxel and gemcitabine, there was a decrease in the density of cancer associated fibroblasts (37). The mechanism of whether nab-paclitaxel truly depletes stromal tissue and the underlying mechanism is still controversial.

The SWOG 1816 phase II/III trials demonstrated that the addition of abraxane to gemcitabine plus cisplatin in advanced biliary tract cancers resulted in a median overall survival of 19.2 months and DCR of 84%, which was an improvement over historical controls (38). There is currently an ongoing phase III trial comparing gemcitabine plus cisplatin with or without abraxane. Recent preliminary data from that trial did not demonstrate a difference between the two groups in median overall survival; however, on an exploratory analysis there may be an improvement in overall survival for patients with locally advanced disease (33).

### Cytotoxic chemotherapy in the neoadjuvant setting

A large proportion of patients present with anatomically resectable disease with high-risk features (e.g., lymphadenopathy, poorly differentiated tumor, vascular invasion). These high-risk features are associated with early recurrence rates and/or metastatic disease. As such, neoadjuvant chemotherapy (gemcitabine and cisplatin) has been explored to downstage patients and/or treat potential microscopic systemic disease. Using a preoperative chemotherapy approach, if the patient progresses on chemotherapy, then the patient can avoid hepatectomy, which may not provide a survival benefit. In a retrospective study of a prospectively collected database, Le Roy et al. compared outcomes of patients who underwent upfront surgery of resectable ICCA versus individuals who underwent neoadjuvant chemotherapy and surgery for locally advanced ICCA (39). Following neoadjuvant chemotherapy, 53% of patients with locally advanced ICCA underwent surgery. There was no difference in overall or recurrence free survival among patients who received preoperative chemotherapy versus upfront surgery. About a third of the downstaged patients had an R0 resection. In a separate retrospective study, Kato et al. reported that 36% of patients with locally advanced ICCA were downstaged with chemotherapy and were able to undergo an operation (40). These patients had longer survival than individuals who could not undergo resection after chemotherapy. There are no published randomized trials evaluating the efficacy of neoadjuvant chemotherapy in ICCA. The phase II NEO-GAP trial did, however, evaluate the use of neoadjuvant gemcitabine, cisplatin, and nab-paclitaxel among patients with high risk ICCA (41). In this study, high risk was defined as tumor size >5cm, multifocal disease, major vascular invasion, or lymphadenopathy. The combination of gemcitabine, cisplatin, and nab-paclitaxel was noted to be safe and did not have a negative impact on surgical outcomes.

### Cytotoxic chemotherapy in the adjuvant setting

The BILCAP phase III clinical trial randomized patients who underwent upfront surgery for ICCA, ECCA, or gallbladder cancer to receive adjuvant capecitabine or observation (42). The capecitabine cohort had an overall survival of 51.1 months compared with 36.4 months in the observation arm. In the long term follow up study, the intention-to-treat analysis confirmed these findings (43). Two additional trials in recent years have failed to demonstrate a survival benefit with gemcitabine-based regimen in the adjuvant setting for biliary tract cancers (44, 45). As such, guidelines still recommend adjuvant capecitabine for patients who undergo upfront resection of ICCA.

### Targeted therapy

Through molecular profiling, genetic aberrations that may contribute to tumorigenesis and cancer progression can be identified. This information can be harnessed to develop targeted therapies. Based on genomic analysis, ICCA and ECCA tumors have different genetic landscapes, further confirming that these tumors are biologically unique and should be treated as separate diseases (46). Fibroblast growth factor receptor (FGFR) and isocitrate dehydrogenase (IDH) genetic aberrations have been identified as the most promising targets in ICCA (47, 48).

### FGFR

**Figure 2:**
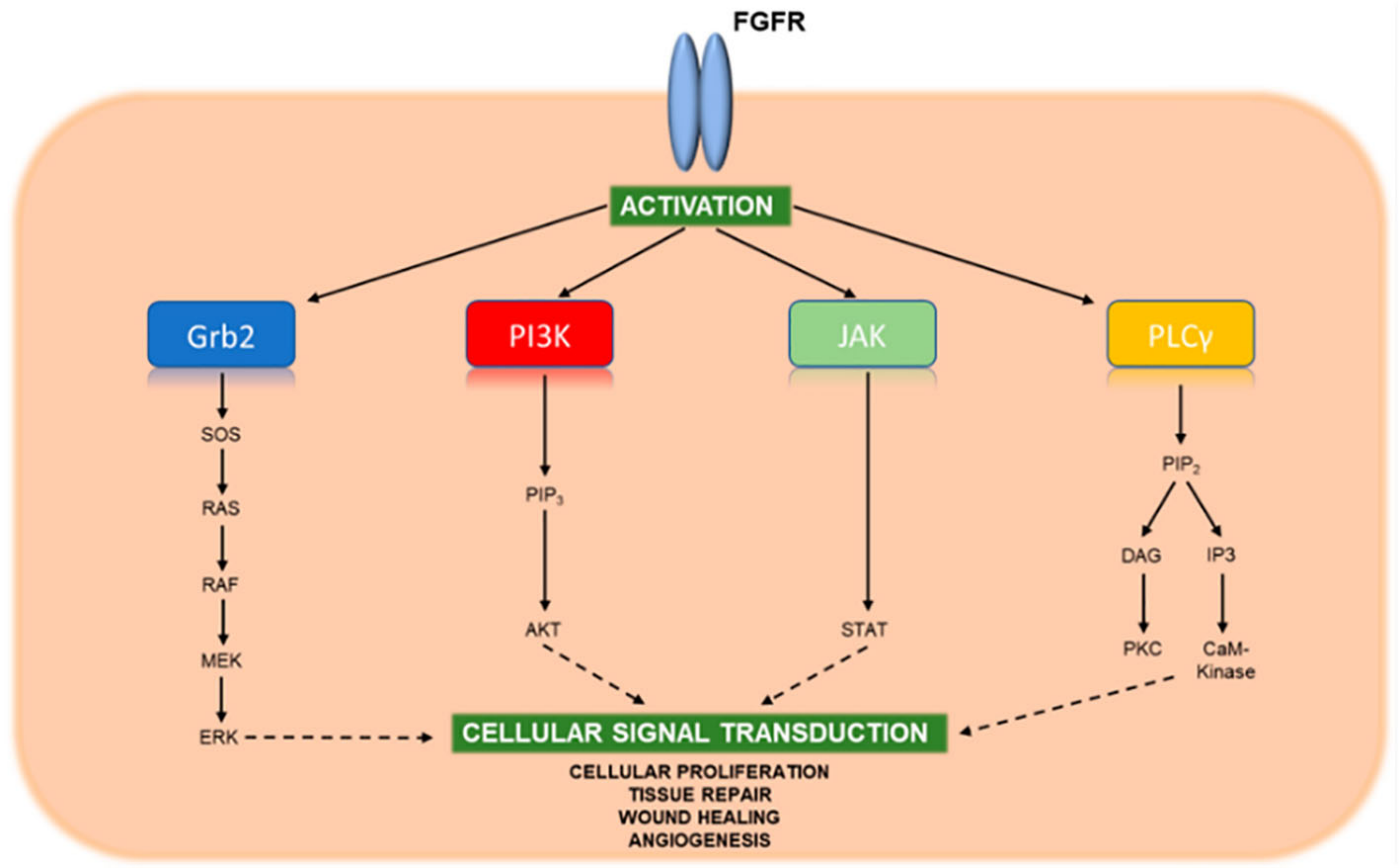
Key signaling pathways of activated FGFR. Upon binding FGF ligands, FGFR molecules dimerize and undergo cross-phosphorylation of tyrosine residues within their activation loops located near the cytoplasmic tail. This phosphorylation activates the kinase domaine, which in turn binds and phosphorylates adaptor proteins of downstream stignaling pathways leading to cellular proliferation, tissue repair, wound healing, and angiogenesis. Reprinted with permission from reference (49).

There are 22 fibroblast growth factors (FGF) and four transmembrane FGFRs. FGF ligands bind the FGFR and stimulate intracellular phosphorylation of the tyrosine receptor kinase domain (**Figure 2**). This pathway sets off a cascade of signaling that induces cell survival and proliferation through the Ras-Raf-MEK-ERK, JAK-STAT, and PI3K-AKT-mTOR pathways (50). Genetic aberrations in the FGFR genes leads to constitutive activation of the receptor and uninhibited cell proliferation and potential carcinogenesis (50). In a study that sequenced 4,853 solid tumors, FGFR genetic aberrations were noted in 7.1% of all cancers and 7% of CCA tumors (51). Within ICCA, FGFR genetic aberrations are found in about 10-15% of tumors (52). The most common FGFR genetic aberration in ICCA is an FGFR2 fusion (52). Pre-clinical studies have demonstrated that knockdown of FGFR2 can inhibit cell growth and colony formation in CCA cells (53).

### FGFR tyrosine kinase inhibitors

Initially, FGFR tyrosine kinase inhibitors (TKI) were non-selective and targeted the conserved ATP-binding domain on the FGFR. This domain is also present on other receptors (e.g. vascular endothelial growth factor receptor (VEGFR) or platelet derived growth factor receptor (PDGFR)) (54). As such, these non-selective TKI at therapeutic levels led to increased toxicity from unintended effects on the other receptors/pathways. The development of second and third generation FGFR inhibitors have improved the ability to select for FGFR, thereby making these agents more potent with an improved safety profile (54, 55).

Infigratinib was the first selective FGFR TKI to demonstrate efficacy in early clinical trials and subsequently was approved for previously treated, advanced/metastatic CCA with an FGFR2 genetic aberration (56, 57). The PROOF-301 trial is currently evaluating infigratinib as a first line therapy option in patients with advanced CCA (NCT03773302). Futibatinib is a selective FGFR TKI that forms an irreversible bond to the receptor (54, 58). The FOENIX-CCA2 trial evaluated the use of futibatinib in 103 patients with previously treated ICCA and an FGFR2 genetic aberration (59). Treatment with futibatinib resulted in an objective response rate (ORR) of 42% and disease control rate (DCR) of 83%. Median progression free survival was 9 months and median overall survival was 21.7 months. Futibatinib is currently approved for the treatment of metastatic ICCA with an FGFR genetic aberration and is being tested as a first line therapy option for ICCA in the FOENIX-CCA3 trial (NCT04093362).

Another promising selective FGFR TKI is pemigatinib. The FIGHT-101 trials studied pemigatinib in patients with previously treated solid tumors irrespective of FGFR status (60). Among patients included in this study, 16.4% had CCA. Pemigatinib was safe and most effective in patients with FGFR genetic aberrations and CCA. The FIGHT-202 trial specifically evaluated pemigatinib’s efficacy in patients with advanced or metastatic CCA, again irrespective of FGFR status (61). Patients without an FGFR genetic aberration did not achieve any response to the treatment, but individuals with FGFR2 fusions or rearrangements had an ORR of 35.5% and median overall survival of 21.1 months. The FIGHT-302 trial is currently comparing pemigatinib to gemcitabine plus cisplatin as first line therapy in patients with FGFR2 genetic aberrations and CCA (NCT03656536). Pemigatinib has been approved by both the United States and Europe for treating advanced or metastatic CCA with an FGFR2 genetic aberration (62). Other FGFR TKI, like erdafitinib and derazantinib have demonstrated some promise in early studies, but results from ongoing trials are still pending (NCT03230318, NCT02699606, NCT04083976).

### IDH

IDH exists in three isoforms with varying expression levels dependent on the tissue type. Both IDH1 and IDH2 are highly expressed in hepatocytes and have been mutated in 20% and 5% of patients with ICCA, respectively (63, 64). IDH1 and IDH2 are involved in a two-step reaction in the Krebs cycle that is crucial for redox homeostasis. While the mechanism for how mutated IDH leads to carcinogenesis is unclear, preclinical studies support that it is due to the accumulation of 2-hydroxyglutarate (2-HG) (65–67). 2-HG competitively binds and inhibits dioxygenase enzymes, which are critical to cell differentiation and metabolism. As such, IDH mutations likely play a role in preventing hepatic progenitor cell differentiation leading to the persistence of stem-like cells that are more prone to oncogenic alterations and tumorigenesis (65, 66, 68). Additionally, 2-HG may contribute to immunosuppression of the tumor microenvironment through inhibition of T-cell proliferation and decreased interferon-γ and IL-2 (65, 69, 70).

### IDH inhibitors

Ivosidenib binds IDH1 and locks it in its inactive form thereby inhibiting the production of 2-HG (71). Early studies established its safety and potential efficacy in patients with previously treated IDH1 mutated CCA (72). In the ClarIDHy clinical trial, ivosidenib was compared with placebo in patients with previously treated advanced or metastatic IDH1 mutated CCA (73). This randomized, double-blind trial included mostly patients with ICCA (91%). Median overall survival for the ivosidenib versus placebo cohort was 10.3 versus 7.5 months, respectively; however, 70% of patients in the placebo arm crossed over to the treatment arm. Taking this into account, the median overall survival was only 5 months for the placebo arm; there was a statistically significant difference between the two cohorts. As such, ivosidenib is now recommended as a second-line therapy for IDH1 mutated CCA (17).

LY3410738 is a selective IDH1 inhibitor that has demonstrated greater potency for inhibiting 2-HG production in the *in vitro* setting (74). LY3410738 is currently being evaluated in a phase I clinical trial in patients with IDH1 mutated solid tumors (NCT04521686) with plans to expand to a phase II trial for patients with CCA that compares LY3410738 combined with gemcitabine and cisplatin in treatment naïve patients to its use as a monotherapy in previously treated patients (74).

### Immunotherapy in ICCA

**Figure 3:**
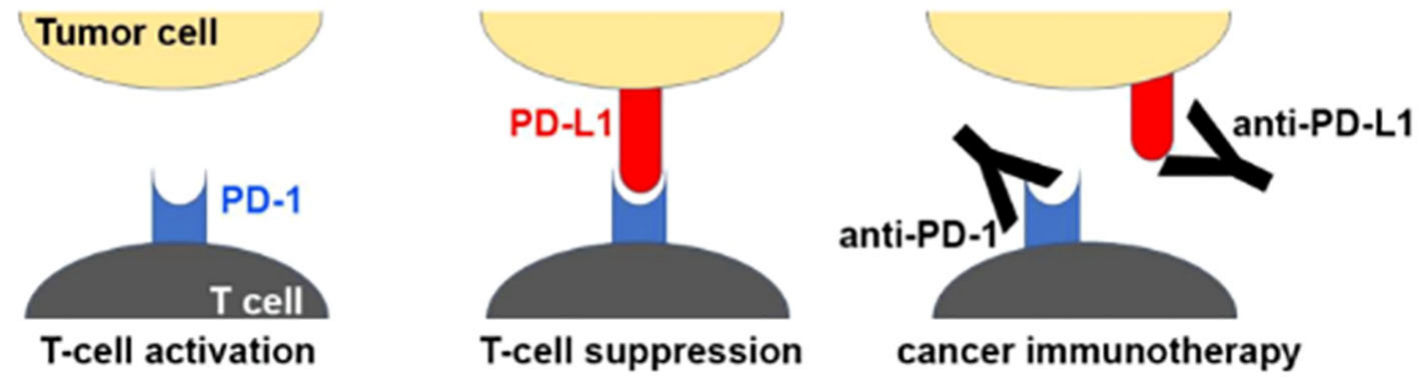
Mechanism of PD1/PD-L1 checkpoint blockade by therapeutic antibodies for cancer immunotherapy. T cell activation is suppressed by the interaction between programmed death 1 (PD-1) on T cells and PD-L1 on tumor cells. Antibody drugs for cancer immunotherapy bind to PD-1 or PD-L1, blocking the PD-1/PD-L1 interaction. Reprinted with permission from reference (78).

Cytotoxic chemotherapy is effective against cancer cells, but unfortunately also exerts its effects on healthy cells, which leads to side effects and safety concerns. Immune checkpoint inhibitors (ICI) have proven to be effective against cancer with a more acceptable safety profile. These medications target immune checkpoints, which are proteins that bind immune cell receptors to inhibit or stimulate the immune system. By upregulating inhibitor proteins and downregulating stimulatory proteins, cancer cells can evade the immune system. ICI restore the immune system’s ability to recognize and kill cancer cells (75–77). Durvalumab is a programmed death ligand-1 (PD-L1) inhibitor (**Figure 3**). The TOPAZ-1 trial demonstrated that the addition of durvalumab to cisplatin and gemcitabine in patients with biliary tract cancers resulted in improved progression free survival and overall survival (79). However, it is important to note that the median overall survival, while statistically significant, was only 12.8 months in the durvalumab cohort versus 11.5 months in the placebo cohort. Durvalumab was approved in 2022 for patients with locally advanced or metastatic biliary tract cancers. A phase II trial evaluated the use of gemcitabine and cisplatin with or without durvalumab in the perioperative setting (neoadjuvant and adjuvant) among patients with biliary tract cancers (80). Neoadjuvant therapy with durvalumab was associated with a likelihood of higher surgical resection, as well as improved survival. There are limited data on neoadjuvant immunotherapy for cholangiocarcinoma, which is an area of ongoing investigation.

Both durvalumab and pembrolizumab inhibit the PD-1/PD-L1 immune checkpoint, but these agents differ slightly in the mechanism of action. Durvalumab binds the ligand (PD-L1), while pembrolizumab binds the T cell receptor PD-1. The randomized, double-blind phase III KEYNOTE-966 trial is currently comparing gemcitabine and cisplatin with either pembrolizumab (PD-1 inhibitor) or a placebo in patients with locally advanced or metastatic biliary tract cancers (NCT04003636) (81). Data recently presented noted that the median overall survival in the pembrolizumab cohort was 12.7 months compared to 10.9 months in the placebo cohort. These data suggest that pembrolizumab may be a potentially new therapy for the treatment of CCA, but the final results of this study are still pending. While difficult to compare clinical trials, preliminary survival data from the KEYNOTE-966 trial using pembrolizumab were comparable to survival data from the TOPAZ-1 trial using durvalumab. In turn, pembrolizumab and durvalumab may have similar effectiveness in the treatment of advanced biliary tract cancers.

## Conclusion and future directions

ICCA remains an aggressive disease with limited treatment options. Curative intent surgery is dependent on appropriately selecting patients who will derive the greatest oncologic benefit and performing a sound operation. While curative intent surgery and adjuvant capecitabine can prolong survival, many patients are diagnosed with late-stage disease or develop recurrence. As such, the last several decades have focused on developing new systemic treatments, like targeted therapy or immunotherapy, that may improve long term outcomes. Coordinated efforts between large centers to share data, expedite accrual for clinical trials, and increase tissue collection for genetic analysis are crucial to moving the field forward.

## Author contributions

SR: Writing – original draft, Writing – review & editing. TP: Writing – review & editing.
